# HDAC inhibitors and immunotherapy; a double edged sword?

**DOI:** 10.18632/oncotarget.2289

**Published:** 2014-07-31

**Authors:** Michiel Kroesen, Paul Gielen, Ingrid C. Brok, Inna Armandari, Peter M. Hoogerbrugge, Gosse J. Adema

**Affiliations:** ^1^ Department of Tumor Immunology, Radboud Institute for Molecular Life Sciences, Radboud University Medical Centre, Nijmegen, The Netherlands; ^2^ Department of Pediatric Oncology, Radboud University Medical Centre, Nijmegen, The Netherlands; ^3^ Princes Máxima Center for Pediatric Oncology, The Bilt, The Netherlands

**Keywords:** HDAC inhibitor, cancer, immunotherapy, immunocombination therapy

## Abstract

Epigenetic modifications, like histone acetylation, are essential for regulating gene expression within cells. Cancer cells acquire pathological epigenetic modifications resulting in gene expression patterns that facilitate and sustain tumorigenesis. Epigenetic manipulation therefore is emerging as a novel targeted therapy for cancer. Histone Acetylases (HATs) and Histone Deacetylases (HDACs) regulate histone acetylation and hence gene expression. Histone deacetylase (HDAC) inhibitors are well known to affect cancer cell viability and biology and are already in use for the treatment of cancer patients. Immunotherapy can lead to clinical benefit in selected cancer patients, especially in patients with limited disease after tumor debulking. HDAC inhibitors can potentially synergize with immunotherapy by elimination of tumor cells. The direct effects of HDAC inhibitors on immune cell function, however, remain largely unexplored. Initial data have suggested HDAC inhibitors to be predominantly immunosuppressive, but more recent reports have challenged this view. In this review we will discuss the effects of HDAC inhibitors on tumor cells and different immune cell subsets, synergistic interactions and possible mechanisms. Finally, we will address future challenges and potential application of HDAC inhibitors in immunocombination therapy of cancer.

## INTRODUCTION

Recent reports have demonstrated synergistic effects of HDAC inhibitors with cancer immunotherapy. Whereas the direct cytotoxic effects of HDAC inhibitors on cancer cells are well known, the effects of HDAC inhibitors on the immune system are less well understood. Here, we review the effects of HDAC inhibitors on immune cell function in relation to anti-tumor immunity. Furthermore, we discuss the mechanisms of HDAC inhibitors in combination with immunotherapy in the treatment of patients with cancer and provide future directions for research.

Only a few gene mutations may lead to the change of a healthy normal cell into a malignant cancer cell. The genetic instability of a developing tumor leads to further accumulation of genomic mutations/alterations but also to changes in the epigenetic code, the epigenome[1]. The epigenome regulates the heritable patterns of gene expression without changing the sequence of the genome[2]. In cancer, alterations to the epigenome may lead to gene expression profiles that support tumorigenesis and thereby play an important role in cancer initiation and progression[3-6]. Epigenetic changes can occur at multiple levels including direct modifications of the DNA itself as well as modifications of the DNA associated histone proteins[7]. Histone proteins can be chemically modified by acetylation, methylation, phosphorylation, and ubiquitination[8]. Hyperacetylation of histone proteins results in increased transcriptional activity, whilst histone hypoacetylation is associated with repression of gene transcription[9]. Hypoacetylation of histones was shown to occur specifically in a variety of human cancer cell lines as well as in primary lymphoma and colon carcinoma tissue samples[10].

Immunotherapy is a promising novel cancer therapy for multiple cancer types, including melanoma and neuroblastoma[11, 12]. Immunotherapy can lead to clinical benefit and active anti-tumor immune responses have been observed in selected patients[13]. However, in most patients, the clinical response is still limited or even absent following immunotherapy[14]. Accumulating evidence indicates that tumors evade immune responses by down regulation of MHC molecules and tumor antigens or active suppression of anti-tumor immune responses at the site of the tumor by creating an immune suppressive tumor microenvironment (TME)[15-18]. Therefore, immunotherapy should best be combined with other therapies in so-called immunocombination therapy to overcome these tumor induced immune escape mechanisms[19].

One potential type of therapies that could be combined with immunotherapy are therapies that target the epigenetic code. Epigenetic alterations are dynamic and generally reversible and for this reason epigenetic manipulation has emerged as an attractive novel treatment for cancer[20]. Small molecule inhibitors were identified that target the enzymes responsible for the deacetylation of histones, the histone deacetylases (HDACs). These so-called HDAC inhibitors, are now regarded as a group of anti-cancer drugs with high clinical potential[9, 21, 22]. Inhibition of HDACs leads to genomic effects through accumulation of acetylated histone proteins, resulting in altered gene transcription[23]. Specifically in cancer cells, the altered gene transcription leads to, amongst others, the activation of and/or sensitization to intrinsic and extrinsic apoptosis pathways[24, 25]. Besides blocking the function of HDACs leading to genomic effects, HDAC inhibitors also modulate the function of many other proteins resulting in non-genomic effects. For example, p53 becomes hyper acetylated upon HDAC inhibitor treatment, resulting in tumor cell apoptosis[26, 27]. Interestingly, tumor cells appear much more sensitive to the induction of apoptosis by HDAC inhibitors than normal cells, although the responsible mechanisms are still not fully understood. Besides changes in gene transcription other possible mechanisms for the tumor specificity of HDAC inhibitors have been suggested, e.g. induction of double-strand DNA breaks[28]. Other reports link the selective sensitivity of cancer cells to HDAC inhibitors relative to normal cells to the disturbed chromatin structure in cancer cells[29].

The classical HDAC inhibitors inhibit the function of one or more of the 11 known zinc-containing HDAC enzymes. The zinc-containing HDAC enzymes can be classified into several Classes: Class I HDAC (HDAC 1,2,3,8), Class IIA (HDAC 4,5,7,9) and Class IIB (HDAC 6,10). Class III HDACs or Sirtuins, have a different catalytic mechanism and are not a target for the classical HDAC inhibitors. The most recently discovered HDAC11 is the only Class IV HDAC[9]. PanHDAC inhibitors inhibit HDACs from Class I, II and IV, while Class specific HDAC inhibitors only inhibit HDACs from either Class I or Class II. More recently, HDAC inhibitors preferentially targeting a single HDAC have been developed[30, 31]. Because each individual HDAC inhibitor has a unique chemical structure and HDAC inhibitory profile, different HDAC inhibitors can cause a large variety of biological effects in cancer cells and in normal cells[32, 33].

Although the effects of HDAC inhibitors on tumor cells have been studied extensively, the exact role of HDAC inhibitors on immune cells and in anti-tumor immunity is just emerging. HDAC inhibitors were previously reported to exhibit strong anti-inflammatory effects[34-36]. Rapamycin, however, previously viewed as a pure immune suppressant drug, was more recently also shown to mediate strong immune stimulating effects as reviewed in[37, 38]. Similarly, a recent study showed that the Class I HDAC inhibitor Entinostat markedly enhanced anti-tumor vaccination[39]. In this review we will discuss the effects of classical pan-, Class I and Class II HDAC inhibitors on tumor cells and immune cells in relation to immunotherapy. Furthermore, we will discuss the potential of combining HDAC inhibitors with immunotherapy as immunocombination therapy for cancer.

### Effects of HDAC inhibitors on tumor cells; immunological consequences

HDAC inhibitors can induce tumor cell death in a specific manner through various mechanisms and with different half maximum inhibitory concentrations (IC50s) as reviewed elsewhere[9, 22, 40]. However, HDAC inhibitors also have profound effects on the remaining viable tumor cells. Here we will discuss the effects of HDAC inhibitors on the cell biology of these surviving tumor cells with a focus on the immunological consequences.

### Effects of HDAC inhibitors on tumor cell recognition by T cells and NK cells

Tumor cell recognition and elimination by tumor specific T cells is, amongst others, dependent on the expression levels of both tumor associated antigens (TAAs) and MHC Class I (MHCI) molecules by the tumor cells. HDAC inhibitors can increase TAA expression by tumor cells. The Class I HDAC inhibitor Depsipeptide enhanced the expression of the tumor antigen gp100 in murine melanoma cells[41]. Combination treatment of Depsipeptide and adoptive transfer of gp100-specific cytotoxic T cells resulted in increased tumor killing by transferred tumor specific CD8 T cells. However, sometimes TAA-expression is downregulated after HDAC inhibitor treatment. The panHDAC inhibitor Valproic acid (VPA) downregulated the expression of the tumor antigen Muc1. Remarkably, it upregulated another tumor antigen NY-ESO-1 in mesothelioma cells *in vitro*[42].

Other reports have shown that HDAC inhibitors upregulate genes involved in the antigen presentation machinery or co-stimulatory molecule expression by tumor cells[43, 44]. The panHDAC inhibitor Trichostatin A (TSA) up-regulated MHCI surface expression in a murine cervical cell line with an impaired antigen-processing machinery[45]. TSA pre-treatment of these tumor cells resulted in more effective lysis of these tumor cells by CD8 T cells *in vitro*. Similarly, TSA was able to increase or induce expression of *TAP-1*, *TAP-2*, *LMP-2*, and *Tapasin* in TAP-expressing and TAP–deficient murine tumor cell lines[46]. TSA treatment of mice bearing TAP-deficient tumors delayed tumor growth due to enhanced tumor cell killing by adaptive immune effector cells. More recently, the panHDAC inhibitor Panobinostat was shown not only to enhance the expression of several TAAs, MHCI and MHCII, but also the expression of co-stimulatory molecules in several human and a murine melanoma cell lines *in vitro*[47]. In addition, effective therapy of B16F10 melanoma bearing mice using Panobinostat was dependent on the presence of the adaptive immune system. In another study, TSA induced MHC Class II expression in murine plasmacytoma cells through activation of the pIII-CIITA promoter, resulting in enhanced proliferation of CD4 T cells *in vitro*[48]. In summary, HDAC inhibitors can modulate TAA expression and many components of the tumor antigen processing and MHC presentation pathway in surviving tumor cells, overall resulting in enhanced tumor cell recognition and killing by tumor specific T cells (Table 1).

NK cells are innate immune cells that exert important anti-tumor effector functions in cancer immunotherapy[49]. The outcome of an interaction between a tumor cell and a NK cell is balanced by the expression of activating and inhibitory ligands by the tumor cell. Several stress-induced activating ligands, like MHC class I-related chain A (MICA) and B molecules (MICB), and UL16-binding proteins (ULBPs), expressed by tumor cells are recognized by the activating NKG2D receptor on NK cells[50]. HDAC inhibitors were shown to increase the expression of activating ligands for this NK cell receptor by tumor cells. The Class I HDAC inhibitor VPA induced the expression of MICA, MICB and ULBPs in human hepatocellular carcinoma cells resulting in enhanced recognition and killing by NK cells *in vitro*[51]. Importantly, in non-malignant primary human hepatocytes, VPA treatment did not induce the expression of these NKG2D ligands. Although the mechanisms for the tumor specific induction of NKG2D ligands remained unclear, this study confirms the selective effect of this panHDAC inhibitor on the expression of NK cell activating ligands by malignant cells. Lopez-Soto et al. showed that TSA increased the expression of ULBPs in epithelial tumor cells by releasing HDAC3 mediated repression on ULBP promotors[52]. Similar effects have been reported for other panHDAC inhibitors when added to osteosarcoma, leukemia and Ewing sarcoma cell lines *in vitro* and to primary myeloid leukemia cells *ex vivo*[53-56]. These studies collectively show that panHDAC inhibitors can increase the expression of activating NKG2D ligands resulting in enhanced tumor cell recognition and elimination by NK cells.

HDAC inhibitors, however, do not always increase tumor cell recognition by immune cells. Fiegler et al. showed that the panHDAC inhibitor Vorinostat and other panHDAC inhibitors down regulated the B7 family member B7-H6, a ligand for the activating NKp30 receptor on NK cells, in multiple human cancer cell lines, both at the mRNA and protein level[57]. The decreased surface expression of B7-H6 resulted in decreased degranulation of primary NK cells in an NKp30 dependent manner. Thus, HDAC inhibitors can also mediate the downregulation of activating ligands for NK cells by tumor cells. Furthermore, HDAC inhibitor induced increased MHCI expression, leading to increased tumor cell recognition by T cells, at the same time will negatively affect NK cell recognition. Besides lymphocytic NK and T cells, macrophages and other myeloid cells are also able to recognize and kill tumor cells directly[58]. However, the effects of HDAC inhibitors on tumor cell recognition by myeloid cells have not been reported to date. We conclude that, in general, HDAC inhibitors lead to enhanced recognition and elimination of tumor cells by effector lymphocytes, but this effect may vary between tumor types and HDAC inhibitors used.

### HDAC inhibitors and immunogenic cell death

Selected chemotherapeutics can induce so-called “immunogenic cell death”, a process in which dying tumor cells can stimulate cellular uptake, activation and cross-presentation by antigen presenting cells (APCs), thereby inducing antitumor T cell responses[59]. Christiansen et al. showed that MC38 colon carcinoma cells, treated with the panHDAC inhibitor Vorinostat, were efficiently taken up by dendritic cells (DCs) *in vitro*[60]. In addition, in other studies, Vorinostat stimulated the release of important mediators of immunogenic cell death, like HMGB1 and ATP, as well as the expression of cell surface Calreticulin, an important ‘eat-me’ signal, by dying tumor cells[61, 62]. However, the precise role of immunogenic cell death upon Vorinostat therapy *in vivo* was not investigated in these studies. AK7 pancreatic carcinoma cells, pretreated with Vorinostat or other cytotoxic drugs, have also been used together with the adjuvant BCG as a vaccine. Only the vaccine consisting of the Vorinostat treated tumor cells was able to inhibit tumor growth upon tumor challenge and resulted in increased CD8 T cell infiltration in this experimental setting[63]. These studies imply that Vorinostat, like a subset of chemotherapeutic compounds, induces a form of cell death with immunogenic properties. It remains to be determined whether other panHDAC or Class specific HDAC inhibitors also induce immunogenic cell death. Furthermore, it will be interesting to compare the potency of Vorinostat and other HDAC inhibitors to induce immunogenic cell death with that of chemotherapeutic agents previously demonstrated to induce immunogenic cell death[64].

### Effect of HDAC inhibitors on immune cells

So far, we discussed the effects of HDAC inhibitors on tumor cell biology and the immunological consequences. In the next paragraphs we will review the effects of HDAC inhibitors on immune cell viability and function and address the mechanisms and critical factors of successful combinations of HDAC inhibitors and immunotherapy *in vivo*. Immune cells can have both pro- and anti-tumor effects, depending on cell lineage and environmental cues. For example, in a recent study by West et al, successful treatment of MC38 colon carcinoma tumors using the panHDAC inhibitors Vorinostat and Panobinostat was fully dependent on the presence of an intact immune system[61].

### Effect of HDAC inhibitors on antigen presenting cells and cytokine production

In order to generate effective and long-lasting immune responses, activation of both the innate and adaptive arms of the immune system is required. APCs, like monocytes, macrophages and DCs are the first innate immune cells to sense danger signals coming from foreign or damaged self[65]. Upon encounter of these signals, APCs become activated and start to recruit and activate other immune cells and thereby initiate antigen specific immunity[66]. DCs are professional APCs and are therefore especially capable of inducing adaptive immunity. Besides signal 1 coming from specific recognition of antigens presented in MHCI and MHCII molecules, co-stimulatory and cytokine signals (signals 2 and 3, respectively) are required to initiate and direct adaptive immune responses[67].

HDAC inhibitors were repeatedly shown to down regulate both co-stimulatory and cytokine signals coming from APCs. The panHDAC inhibitors Vorinostat and TSA profoundly down-regulated genes involved in co-stimulation and the production of cytokines in murine bone marrow derived macrophages (BMDM) and dendritic cells (BMDC)[68, 69]. Upon treatment with TSA, surface expression of CD40, CD80, CD86 and CCR7 as well as the production of pro-inflammatory cytokines IL-6, IL-12 and TNF-α was down regulated in BMDC[69]. In contrast, TSA and the panHDAC inhibitor Panobinostat increased IL-12 production in peritoneal elicitated macrophages, which was associated with a downregulation of the anti-inflammatory cytokine IL-10[70]. One explanation for these different findings could be that the timing of HDAC inhibitor exposure differed between these studies: in the first studies the APCs were exposed to the HDAC inhibitor one hour before the immune stimulus, whereas in the latter study the HDAC inhibitor and the immune stimulus were administered together. These observations suggest that the pre-activation of these APCs leads to differential effects upon HDAC inhibitor treatment. Also, in contrast to the panHDAC inhibitor TSA, the Class I specific HDAC inhibitor Entinostat did not down regulate IL-12 mRNA in BMDM, suggesting Entinostat may prevent the reduced production of IL-12 in these APCs[71]. In addition, HDAC6 was required for the production of IL-10 in murine APCs, suggesting panHDAC and Class II HDAC inhibitors may downregulate IL-10 production by APCs[72].

**Figure 1:**
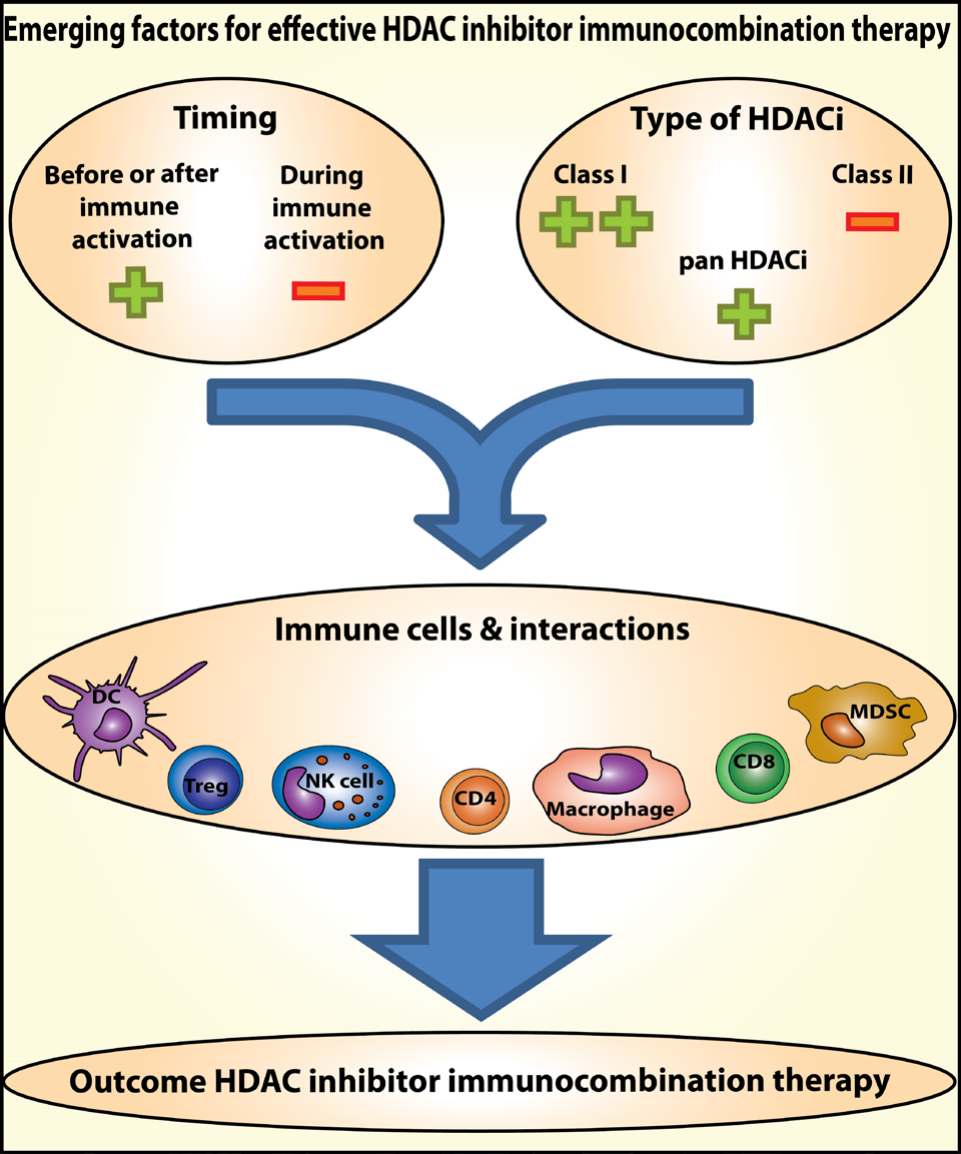
Emerging factors for effective HDAC inhibitor immunocombination therapy Timing of HDAC inhibitor administration and the type of HDAC inhibitor used, determine the effect on the various types of immune cells and their interactions. Therefore, these are emerging factors determining the outcome of combinations of HDAC inhibitors with immunotherapy in the treatment of cancer.

Support for a role of individual HDACs in the initiation of inflammatory cytokine production by APCs comes from studies using HDAC3^−/−^ murine BMDM. These cells were shown to be incapable of a pro-inflammatory gene response when stimulated with LPS[73]. The lack of expression of pro-inflammatory genes could be largely explained by a lack of IFN-β expression in these macrophages. This was most probably due to the constitutive over expression of Cox-1 in the se macrophages, which interfered with pro-inflammatory signaling cascades. In addition, other studies have reported on panHDAC inhibitor mediated downregulation of systemic inflammation by reducing pro-inflammatory cytokine production in mice[34, 35, 74-77]. Similar suppressive effects of HDAC inhibitors on pro-inflammatory cytokine production were also reported for human macrophages and DCs[76, 78-81]. In human monocyte derived DCs, the reduced cytokine production upon HDAC inhibitor exposure was suggested to be mediated through decreased NF-kB and type I interferon signaling as suggested by the reduced nuclear translocation of NF-kB RelB, IRF-3 and IRF-8[82]. Similarly, upon exposure to Panobinostat, RelB was down-regulated in a dose-dependent manner in human monocyte derived DCs[83]. Cytokine production by immune cells was also reduced upon administration of the panHDAC inhibitor Givinostat to healthy human subjects[84]. Givinostat treatment reduced the pro-inflammatory cytokine production by endotoxin stimulated PBMC*,* four hours after Givinostat administration *ex vivo*. Twelve hours after Givinostat administration, however, cytokine production by the PBMC had already returned to baseline levels, suggesting a transient reduction in cytokine production. This observation was in agreement with the half-life of Givinostat of around 6 hours as determined in the same study. This transient reduction of pro-inflammatory cytokine production following Givinostat treatment, could imply that the timing of HDAC inhibitor treatment may be crucial when combining HDAC inhibitors with immunotherapy (Figure 1). In this respect, short pulses of Givinostat might circumvent prolonged immune suppression in immunocombination therapy regimens. Overall, these data suggest that HDAC inhibitors suppress macrophage and DC functions in terms of reduced expression of co-stimulatory molecules and reduced production of pro-inflammatory cytokines.

The effect of HDAC inhibitors on the (cross-) presentation of antigens by APCs has not been studied extensively. TSA reduced the capacity of murine BMDC to induce T cell proliferation in mixed-lymphocyte-reactions, but the contribution of antigen (cross-)presentation was not investigated[85].

Besides down-regulating pro-inflammatory cytokines, HDAC inhibitors can also reduce the production of pro-tumorigenic soluble factors or cytokines. Vorinostat reduced the production of the tumorigenic factors Nitric Oxide (NO) and M-CSF & MMP-9 by murine peritoneal macrophages and primary mammary tumor cells, respectively[86]. Administration of Vorinostat delayed tumor onset in a spontaneous mammary tumor model, which was associated with reduced numbers of tumor infiltrating macrophages. Similarly, several panHDAC inhibitors reduced the production of the tumorigenic cytokine macrophage migration inhibitory factor (MIF) in several human cancer cell lines as well as in mouse blood[87]. These studies suggest that the production of tumorigenic cytokines or other soluble factors by myeloid cells and tumor cells are also reduced upon panHDAC inhibitor treatment. The net outcome of HDAC inhibitor treatment on anti-tumor immune responses will likely be a delicate balance in the production of pro- and anti-inflammatory cytokines and other soluble factors in the blood, lymphoid organs and, more importantly, locally in the tumor.

### Effect of HDAC inhibitors on effector lymphocytes

CD4 T cells are essential in the induction of adaptive anti-tumor immunity by maintaining and skewing immune responses[88-90]. HDAC inhibitors were reported to have inhibitory effects on CD4 T cell viability and function. The panHDAC inhibitor TSA inhibited PMA/Ionomycin induced NF-kB nuclear translocation in murine CD4 T cells after 8 hours and impaired their viability after 20 hours *in vitro*[91]. TSA also potently decreased the antigen specific proliferation of murine CD4 T cells, which was associated with upregulation of the cyclin-dependent kinase inhibitor 1 p21^Cip1^[92, 93]. In human CD4 T cells, Vorinostat had limited to no effect on viability and the expression of genes involved in proliferation, differentiation, and apoptosis after a 24 hour exposure period[94]. These opposite observations may be species related or due to intrinsic differences between the panHDAC inhibitors used in these studies. Indeed, the panHDAC inhibitor TSA has a higher inhibitory potency for most of the individual HDACs compared to Vorinostat, which could explain the more pronounced effects of TSA in the murine CD4 T cells[95-97]. In patients treated with the Class I HDAC inhibitor Romidepsin, the percentages of CD4 and CD8 T cells in patient's blood were decreased by around 50%[98]. In a study by Schmudde et al, Vorinostat affected the proliferation and function of naïve human PBMC and T cells, but not of IL-2 pre-activated PBMC or previously primed T cells[99]. These studies suggest that pan- and Class I HDAC inhibitors reduce the viability and function of naive CD4 T cells, thus hampering the induction of anti-tumor T cell responses. The inhibitory effect of HDAC inhibitors, however, seems less severe or absent following activation of these CD4+ lymphocytes. Therefore, the timing of HDAC inhibitor administration in combination with immunotherapy could critically determine the outcome of immunocombination therapy, combining HDAC inhibitors with immunotherapy (Figure 1).

For effective anti-tumor immunity the function of cytotoxic CD8 T cells is of crucial importance[13]. Several studies suggest HDAC inhibitors enhance the function of this CD8+ T cell subset. Treatment of mice with the panHDAC inhibitor Panobinostat resulted in increased serum levels of IFN-γ and TNF-α and an accelerated graft-versus-host disease, which was associated with higher numbers of CD8 T cells in the affected organs[100]. Strikingly, in murine CD8 T cells, the panHDAC inhibitor TSA mimicked the effect of the pro-inflammatory cytokines IL-12 and IFN-α at the level of gene expression[101]. TSA up-regulated genes involved in CD8 T cell activation and memory similar to those induced by these pro-inflammatory cytokines. This finding suggests that part of the immune inhibitory effect of HDAC inhibitors on pro-inflammatory cytokine production by APC may be compensated by the direct induction of a set of genes in these effector lymphocytes. Murine memory CD8 T cells were capable of producing IFN-γ only when CD4 T cell help was present during priming[102]. Interestingly, only in these CD8 T cell memory cells, the IFN-γ locus was hyper acetylated, allowing for the rapid production of IFN-γ. More recently, the function of exhausted CD8 T cells in chronic viral infection was shown to be restored upon treatment with panHDAC inhibitors[103]. The effect of Class II HDAC inhibitors on CD4 and CD8 T cells have not been reported to date. These studies collectively suggest that panHDAC inhibitors have the ability to stimulate CD8 T cell activation and function.

Although HDAC inhibitor treated tumor cells are more efficiently recognized by effector NK cells, not much is known regarding the effects of HDAC inhibitors on NK cell function itself. Ogbomo et al. first showed that the Class I HDAC inhibitor VPA and the panHDAC inhibitor Vorinostat inhibited the proliferation and cytotoxic capacity of human NK cells, treated with IL-2 and the HDAC inhibitor simultaneously[104]. The NK cells down regulated the expression of activating receptors on the cell surface upon Vorinostat exposure. In another study, upon exposure to similar concentrations of Vorinostat, NK cells were still able to degranulate upon co-culture with tumor cells[99]. The NK cells from the latter study, however, had received the activating cytokine three days prior to the addition of the HDAC inhibitor and were thus pre-activated. These studies imply that IL-2 pre-activated NK cells retain their functionality whereas NK cells receiving IL-2 simultaneous with the HDAC inhibitor show reduced functionality. In a more recent study, the Class I HDAC inhibitor Entinostat increased the expression of the activating receptor NKG2D in IL-2 and IL-21 activated as well as in freshly isolated human NK cells. Entinostat also increased the expression of NKG2D ligands on human tumor cell lines, but not on normal cells, in this study[105]. Furthermore, Entinostat treated NK cells showed increased cytotoxicity upon co-culture with tumor cells and the combination of Entinostat treatment and adoptive NK cell transfer resulted in synergistic inhibition of tumor growth *in vivo*. Thus, the Class I HDAC inhibitor Entinostat increased activating ligand expression by tumor cells as well as NK cell cytotoxicity. The effects of Class II HDAC inhibitors on NK cells have not been reported to date. These studies suggest that panHDAC inhibitors have distinct effects on NK cells, at least partially depending on their activation status, whereas Class I HDAC inhibition seems to enhance NK cell function.

In conclusion, HDAC inhibitors can either inhibit or promote effector lymphocyte function depending on the cell type, activation status and the type of HDAC inhibitor used.

### Effect of HDAC inhibitors on regulatory immune cells

After induction of effective immunity through activation of both innate and adaptive arms of the immune system, feedback loops are in place to control the ongoing inflammation. Specialized immune cells like CD4+ regulatory T cells (Treg) and specific subsets of regulatory myeloid cells can actively dampen immune responses. Tumors actively recruit Treg, myeloid derived suppressor cells (MDSC) and tumor associated macrophages (TAM)[106]. These immune cells all contribute to an immune suppressive tumor microenvironment (TME), abrogating anti-tumor immune responses.

Several panHDAC inhibitors were shown to promote the expansion and function of CD4+ Treg in multiple mouse studies, often after prolonged daily administrations of panHDAC inhibitors[107-111]. HDAC 6 and HDAC9, both Class II HDAC enzymes, were shown to be expressed by murine CD4+FoxP3+ Treg and acted as inhibitors of the suppressive function of these regulatory immune cells[107, 112, 113]. HDAC9^−/−^ mice showed increased numbers of Treg with increased suppressive capacity[112]. In line with these observations, exposure to Class II HDAC inhibitors was repeatedly shown to directly enhance the suppressive function of murine Treg[113-115]. These studies demonstrate that Class II HDAC enzymes are important regulators of Treg function and that Class II HDAC inhibition results in increased Treg functionality. The effect of Class I HDAC inhibition on Treg proliferation and function is less clear. The Class I HDAC inhibitor Entinostat enhanced Treg numbers and FoxP3 expression by Treg[116, 117]. In another study, however, the numbers of Treg were equal and FoxP3 expression by the Treg was down-regulated following Entinostat treatment, leading to improved anti-tumor vaccination *in vivo*[118]. In addition, Bridle et al. showed both reduced Treg numbers as well as reduced FoxP3 expression in Treg upon Entinostat treatment[39]. These studies collectively indicate that pan- and Class II HDAC inhibitors enhance Treg numbers and function, whereas Class I HDAC inhibitors show more complex effects on Treg, including decreased numbers and function, that need to be studied in more detail. In this respect, Class I HDAC or panHDAC inhibitors might be more suited to combine with immunotherapy than Class II HDAC inhibitors (Figure 1).

MDSC are immature myeloid cells that can actively suppress T cell responses and contribute to an immune suppressive TME[106, 119]. Murine bone marrow precursor cells cultured for 7 days in the presence of GM-CSF & TSA or GM-CSF alone, revealed striking differences in myeloid cell differentiation. The presence of the panHDAC inhibitor TSA throughout this culture period resulted in the accumulation of a pool of undifferentiated myeloid cells. These cells were CD11b(+)Ly6C(+)F4/80(int)CD115(+) and showed immune suppressive properties *in vitro,* thus mimicking MDSC[120]. Treatment of naïve mice with GM-CSF and TSA resulted in a similar accumulation of CD11b(+)Gr1(+) cells in the spleens of these mice, showing immune suppressive activity *ex viv*o. Youn et al. showed that the Class I HDAC inhibitor VPA could differentiate tumor induced MDSC into macrophages and DC following *in vitro* culture[121]. These MDSC were isolated from the BM of tumor bearing mice and cultured with GM-CSF and tumor conditioned medium in the presence or absence of VPA. Thus, panHDAC inhibition affects myeloid cell differentiation from precursors towards MDSC, whereas Class I inhibition directs MDSC to more differentiated macrophages and DC. In the latter study, the Rb1 gene was shown to regulate the differentiation from MDSC to macrophages and DC. Rb1 expression in turn was regulated by HDAC2, showing a role for HDAC2 in MDSC differentiation. The effects of Class II specific HDAC inhibitors on MDSC have not been reported.

TAM expressing low levels of MHCII also accumulate in tumors and are associated with tumor progression[122]. Similarly to MDSCs, TAM have been reported to be sensitive to the Class I HDAC inhibitor VPA and the panHDAC inhibitor TSA, resulting in restoration of MHC class II expression, reversal of immune suppression and delayed tumor growth[123, 124]. The effects of Class II specific HDAC inhibitors on TAM have not been reported.

Overall, the available data suggest that the effect of HDAC inhibitors on regulatory immune cells differ between the immune cell type studied, the differentiation status and the HDAC inhibitor used. The precise effects of HDAC inhibitors on myeloid cells in cancer, like MDSC and TAM, deserve further exploration.

**Table 1:** Overview of the observed effects of HDAC inhibitors on tumor cells and immune cells

	HDAC inhibitor (class)	Observations	References
Tumor cells			
	Depsipeptide (Class I) Valproic Acid (panHDAC) Trichostatin A (panHDAC) LAQ824 (panHDAC) Panobinostat (panHDAC)	TAA↑ MHCI↑ MHCII↑ Co-stimulatory molecules↑ Recognition by T cells↑	[41-43, 45-48, 126]
	Valproic Acid (panHDAC) Trichostatin A (panHDAC)	Expression NKG2D ligands/ Recognition by NK cells↑	[51-56]
	Vorinostat (panHDAC)	Immunogenic cell death↑	[60, 61, 63]
Effector lymphocytes			
CD4 T cells	Trichostatin A (panHDAC) N-butyrate(panHDAC) Scriptaid (panHDAC) Vorinostat (panHDAC) Romidepsin (Class I)	Viability↓ Proliferation↓ Pro-inflammatory cytokines↓	[39, 91-93, 98, 99, 126]
	Vorinostat (panHDAC)	Viability=	[94]
Activated CD4 T cells	Vorinostat (panHDAC)	Viability= Cytotoxicity=	[99]
CD8 T cells	Panobinostat (panHDAC) Trichostatin A (panHDAC)	Pro-inflammatory cytokines↑ Cytotoxicity↑ Memory function↑	[39, 100, 101, 126]
NK cells	Valproic Acid (panHDAC)	Proliferation↓ Cytotoxicity↓	[104]
	Entinostat (Class I)	Cytotoxicity↑	[105]
Activated NK cells	Vorinostat (panHDAC)	Cytotoxicity =	[99]
APC / Cytokine production			
Macrophages /DC	Trichostatin A (panHDAC) Vorinostat (panHDAC) LAQ824 (panHDAC) Panobinostat (panHDAC) Valproic Acid (panHDAC) Entinostat (Class I)	Co-stimulatory molecules↓ Pro-inflammatory cytokines↓ APC function↓	[68, 69, 76, 78-83, 85]
Cytokines / inflammation	Vorinostat (panHDAC) Givinostat (panHDAC)	Pro-inflammatory cytokine production↓ Inflammation ↓	[34, 35, 74, 76, 77, 84]
Macrophages Tumor cells	Vorinostat (panHDAC) Trichostatin A (panHDAC) Valproic Acid (panHDAC)	Tumorigenic sol. factors / cytokines↓	[86, 87]
Regulatory Immune Cells			
Treg	Trichostatin A (panHDAC) Vorinostat (panHDAC) Valproic Acid (panHDAC) Tubacin (Class II) Entinostat (Class I)	Cell numbers↑ FoxP3 expression↑ Immune suppressive capacity↑	[107-113, 116, 117]
	Entinostat (Class I)	Numbers =/↓ FoxP3 expression↓ Immune suppressive capacity↓	[39, 118]
Bone marrow cells	Trichostatin A (panHDAC) Vorinostat (panHDAC)	Differentiation↓ MDSC↑ Macrophages/DC ↓	[120]
MDSC	Valproic Acid (panHDAC)	Differentiation↑ MDSC↓ Macrophages/DC↑	[121]
TAM	Trichostatin A (panHDAC) Valproic Acid (panHDAC)	MHCII expression↑	[123, 124]

### HDAC inhibitors in immunocombination therapy *in vivo*

HDAC inhibitors can impact the immune cascade by influencing different cell types in various life-cycle stages including activation, differentiation, and proliferation. The complex interactions of immune cells in this cascade makes the influence of HDAC inhibitors on the overall outcome of an immune response difficult to predict. In the tumor setting, the growing tumor influences the various immune cells locally and systemically, which further increases the complexity. Thus, the effect of HDAC inhibitor treatment combined with immunotherapy should be investigated by the use of autologous and immunocompetent preclinical models.

As can be concluded from previous sections, HDAC inhibitors can have immune suppressive as well as immune stimulating effects *in vitro*. There are not many studies reporting on detrimental effects of HDAC inhibitors in combination with cancer immunotherapy *in vivo*. HDAC inhibitors, however, have been used to limit cytokine production and immune damage in autoimmune diseases, for example in rheumatoid arthritis[78, 125]. The few studies that have reported synergistic effects of treatments combining HDAC inhibitors and immunotherapy in the treatment of cancer, will be discussed here.

The panHDAC inhibitors Vorinostat or Panobinostat showed a synergistic effect in combination with the immune cell stimulating antibodies anti-CD40 and anti-CD137 in immunocompetent models of mammary, renal and colon carcinoma[60]. This synergistic inhibition of tumor growth was highly dependent on CD8 T cells. Vo et al. demonstrated that the panHDAC inhibitor LAQ824 potentiated both adoptive transfers of tumor specific T cells as well as a prime/boost vaccination scheme in mice bearing B16 melanoma tumors[126]. The authors showed that the adoptively transferred T cells were more abundant in the tumor when LAQ824 was co-administered. Similar to what has been reported *in vitro*[99], naïve T cells were more sensitive to LAQ824 mediated cell death *in vivo*, suggesting a survival advantage of the transferred CD8 T cells. In addition, upon LAQ824 treatment, the tumor cells expressed higher levels of the tumor antigen gp100 and MHCI presenting molecules, implying enhanced recognition by the tumor specific T cells. The adoptively transferred T cells in LAQ824 treated mice produced higher levels of IFN-γ upon re-stimulation *ex vivo,* indicating also direct enhancement of CD8 T cell function by this HDAC inhibitor. In addition to the panHDAC inhibitor LAQ824, also the Class I inhibitor Entinostat showed synergistic anti-tumor effects when combined with IL-2 in mice bearing established RENCA tumors[127]. This synergistic effect was also dependent on the presence of CD8 T cells. More recently, Bridle et al showed that administration of Entinostat enhanced tumor specific T cell function only when Entinostat was given at the time of the booster vaccination, but not at the prime vaccination[39]. Surprisingly, the tumor specific CD8 T cell expansion was not enhanced directly by Entinostat in this study. Instead, Entinostat enhanced functionality of the tumor specific CD8 T cells by creating a prolonged state of lymphopenia following vaccination. The selective elimination of unwanted precursor lymphocytes from the BM resulted in tumor specific CD8 cytotoxic lymphocytes exhibiting enhanced functionality. This study elegantly shows that the timing of HDAC inhibitor treatment is essential for the combination with immunotherapy in order to boost anti-tumor immune responses. Taken together, these studies indicate that carefully designed regimens of HDAC inhibitor treatment and immunotherapy have the potential to be synergistic in the treatment of cancer. The underlying mechanisms of these successful immunocombination therapies are, however, complex and diverse and may include direct tumor cell killing, reprogramming of the tumor microenvironment as well as different effects on innate and adaptive immune cells like depletion of bystander lymphocytes and activation of effector lymphocytes.

## CONCLUSIONS AND FUTURE DIRECTIONS

Although HDAC inhibitors have negative/detrimental effects on immune cell viability and function, increasing evidence also supports a rationale to combine HDAC inhibitors with immunotherapy to obtain synergistic anti-tumor effects. HDAC inhibitors induce tumor cell specific apoptosis already resulting in tumor debulking. Selective elimination of tumor cells also reduces the tumor induced immune suppression and makes the tumor more accessible for immune cells. In addition, HDAC inhibitors can increase tumor cell recognition by NK and T cells. More recently, it was shown that HDAC inhibitors can have both stimulatory as well as detrimental effects on immune cell viability and function, depending on cell type and activation status. Timing is therefore emerging as a crucial factor in obtaining synergistic effects with immunotherapy (Figure 1). For example, based on present data, the administration of HDAC inhibitors should take place following immune activation/CD8 T cell priming, as activated lymphocytes seem less affected and CD8 T cells become more activated by HDAC inhibitors. Besides timing, the Class of the HDAC inhibitor is very important. Inhibition or downregulation of Class II HDACs enhanced Treg numbers and function, whereas the Class I HDAC inhibitor Entinostat enhanced NK cell and CD8 T cell functions. Finally, due to the vast complexity of molecular and cellular events, rational combination therapies of immunotherapy with HDAC inhibitors should be designed and tested using autologous and immunocompetent preclinical models, to elucidate the complex underlying mechanisms. Understanding the mechanisms of such synergistic combinations will be instrumental to efficiently translate these findings into effective immunocombination therapies for cancer patients.

## Abbreviations

APC: Antigen presenting cells
BM: Bone marrow
BMDM: Bone marrow derived macrophage
BMDC: Bone marrow derived dendritic cell
CIITA: Class II trans-activator
DcR3: Decoy Receptor 3
DC: Dendritic cells
FasL: Fas ligand
HAT: Histone acetylases
HDAC: Histone deacetylases
LPS: Lipopolysaccharide
mAb: monoclonal antibody
MDSC: Myeloid derived suppressor cells
MICA: MHC class I-related chain A
MICB: MHC class I-related chain B
MIF: Migration inhibitory factor
NK cell: Natural Killer cell
NO: Nitric oxide
PBMC: Peripheral blood mononuclear cells
TAA: Tumor associated antigen
TAM: Tumor associated macrophage
TME: Tumor microenvironment
TRAIL: Tumor necrosis factor-related apoptosis-inducing ligand
Treg: T regulatory cells
TSA: Trichostatin A
ULBP: UL16-binding proteins
VPA: Valproic Acid
